# Higher dimensional Bianchi type-I string cosmological model in f(R) theory of gravity

**DOI:** 10.1016/j.heliyon.2021.e08063

**Published:** 2021-09-23

**Authors:** Ahmed M. Al-Haysah, A.H. Hasmani

**Affiliations:** a Department of Mathematics, Faculty of Education and Sciences-Rada'a, Albaydha University, Albaydha, Yemen; b Department of Mathematics, Sardar Patel University, Vallabh Vidyanagar-388120, Gujarat India

**Keywords:** N-dimensional Kaluza-Klein cosmology, Cosmic string, f(R) theory of gravity

## Abstract

In this paper, we have studied Bianchi type-I string cosmological model by combining Kaluza-Klein (KK) theory and f(R) theory of gravity which is an extension of 5-dimensional KK string cosmological models. We have used equation of state in the form of p-string or Takabayasi string given by ρ=(1+ω)λ, where *ρ* and *λ* denote the rest density of energy cloud of strings, and the tension density of the system of strings, respectively and *ω* is a constant. In order to get physically significant and viable solution various forms of the function f(R) are assumed, in this paper we assume $f(R)=R+\alpha R^{2}$ (e.g. Astashenok et al. (2017) [1]), where *α* is real number. Some physical and geometrical properties of the model are also discussed.

## 1 Introduction

The 3-dimensional space and time were generalized in 1909 by Lorentz to 4-dimensional space-time, which means the general relativity could be built clarifying gravity with pure geometry.

Nowadays, the studies of cosmologies with more than 4-dimensions gain great importance. These higher dimensional scenarios are built on various KK universe. In terms of utility, KK universe are employed in a way to combine interactions of measurement along with gravity. Here the extra dimensions perform a fundamental physical role and its obscurity is usually clarified by the assumption that they are constrained to compact space with an extremely small length in scale. In 1921, Kaluza's [2] notion was published and he used another dimension to build a more general theory and extended it by Klein in 1926.

Originally, the theory of KK is a result of one extra distinct dimension (four space & one-time) with one interaction (5-dimensional gravity). We have to find the suitable metric tensor for higher-dimensional in f(R) theory of gravity.

The Kaluza-Klein universe was carried out to explore both Maxwell's electromagnetic theory and Einstein's gravitational theory by including the fifth dimension [3].

In view of unifying electromagnetic theory and Einstein's relativity, an additional fifth dimensional was proposed by Kaluza [2]. The new theory received a criticism that it is a vacuum five-dimensional theory which is constructed in the presence of an electromagnetic field. It was further taken into consideration and hence Kaluza opined that general relativity (GR) is not adapted just extend 4-dimensional space to 5-dimensional with having physical relevance to the extra-dimensional. Further, Klein (1926) [4] has affirmed the compactification of the fifth dimension. In a study carried out by Chodos and Detweiler (1980) [5], they revealed that in their 5-dimensional models the additional dimension diminishes the cosmic development.

The unification of electromagnetism and highlighted by Gross and Perry (1983) where they obtain Soliton solutions. Moreover, they made it clear that the difference of the gravitational and inertial masses is caused by the destruction of Burkeff's theorem in KK universe, which is considered to be the principle of equivalence (PoE). Other scholars such as Leon (1988) [6], Chi (1990) [7], Liu (1994) [8], Coley (1994) [9], Ghate (2014) [10], and Trivedi (2021) [11] have surveyed higher dimensional cosmological models in different alternatives to GR. The problem of string cloud and domain walls with the quark model was investigated by Adhav et al., (2008) [12], and the set up of higher dimensional KK theory was successful there.

The phenomenon of string theory was developed to describe events at the early stages of the evolution of the universe. The belief that a strings are seen as an important source for density perturbations that are needed to form large scale structures in the universe which discussed by Vilenkin (1985) [13] and Letelier (1983) [14] in GR. Relativistic string models in the context of Bianchi models have been obtained by Krori et al. (1990) [15]. Bhattacharjee and Baruah (2001) [16] studied the problem of cosmic strings in Bianchi type cosmologies with a self-interacting scalar field.

As an extension to the general theory of gravity f(R) theory is one of the examples. This theory is a generalized version of teleparallel gravity in which the Weitzenbock connection is used instead of Levi- Civita connection. Copeland et al. (2006) [17] have given a complete review of f(R) theory. Myrzakulov (2011) [18] has shown that the acceleration in the expansion of the universe is better understood by f(R) gravity model. In reality, f(R) theory is an extension of standard Einstein-Hilbert action involving a function of the Ricci scalar *R* either linear or non-linear in standard Einstein-Hilbert Lagrangian. The gravitational field equations of f(R) theory are obtained from the Einstein-Hilbert type variational principle [19].

Some energy conditions are analyzed with one of popular models of f(R) gravity, like $f(R)=R+\alpha R^{2}$ where *α*, is constant. There are several models or functional forms of f(R) proposed in the literature, see Avik et al. (2021) [20] for more details. The viability of f(R) models was constrained by several observational data see Capozziello (2006) [21]. It was shown that astrophysical structures like massive neutron stars which cannot be addressed by GR, that can be solved by the higher-order curvature of f(R) gravity, see Astashenok et al. (2017) [1] for more details. The equations of motion of f(R) gravity have higher degrees and provide considerable solutions that are different from general relativity.

In this research article, the higher-dimensional KK string cosmological model has been studied in f(R) theory of gravity, which is the extension of the 5-dimensional KK string cosmological model Nambu., (1983) [14]. To get the desired solution, the equation of state (EoS) for Nambu string assumed by ρ=λ has been taken into account.

## 2 The metric and the field equation

The spatially homogeneous and anisotropic (SHA) Bianchi type-I cosmological model with n-dimensional KK metric of gravitation is given by the metric (see Adhav et al. (2010) [22], Ladke et al. (2014) [23] for more details)

(1) $$ds^{2}=dt^{2}-A_{1}^{2}\sum_{i=1}^{n-2}dx_{i}^{2}-A_{2}^{2}dx_{n-1}^{2},$$

where $A_{1}$ and $A_{2}$ are called cosmic scale factors which are functions of time *t* only. The corresponding Ricci scalar is

$$R=2\left[(n-2)\frac{\overset{¨}{A_{1}}}{A_{1}}+(n-2)\frac{\overset{˙}{A_{1}}\overset{˙}{A_{2}}}{A_{1}A_{2}}+\frac{\left(n-2)(n-3\right)}{2}{\left(\frac{\overset{˙}{A_{1}}}{A_{1}}\right)}^{2}+\frac{\overset{¨}{A_{2}}}{A_{2}}\right],$$

where an overhead dot $\left({}^{\text{.}}\right)$ refers to derivative with respect to time *t*. For the metric (1), the determinant of the metric tensor *g*, spatial volume *V*, average scale-factor a(t), Hubble parameter *H*, deceleration parameter *q*, and the average anisotropy parameter $\overset{¯}{A}$ are

$$\begin{array}{cc} g & =-A_{1}^{2(n-2)}A_{2}^{2},\\ V & =\sqrt{-g}=a^{n-1}=A_{1}^{\left(n-2\right)}A_{2}, \end{array}\;\;\begin{array}{cc} a(t) & =A_{1}^{\frac{n-2}{n-1}}A_{2}^{\frac{1}{n-1}}, \end{array}$$

$$q=-\frac{\overset{¨}{a}}{aH^{2}}=\frac{(n-2)\left({\left(A_{2}{\overset{˙}{A}}_{1}(t)-A_{1}{\overset{˙}{A}}_{2}\right)}^{2}-(n-1)A_{1}A_{2}^{2}{\overset{¨}{A}}_{1}\right)}{{\left((n-2)A_{2}{\overset{˙}{A}}_{1}+A_{1}{\overset{˙}{A}}_{2}\right)}^{2}}-\frac{(n-1)A_{1}^{2}A_{2}{\overset{¨}{A}}_{2}}{{\left((n-2)A_{2}{\overset{˙}{A}}_{1}+A_{1}{\overset{˙}{A}}_{2}\right)}^{2}},H={\left(lna\right)}_{;t}=\frac{\overset{˙}{a}}{a}=\frac{1}{n-1}\sum_{i=1}^{n-1}H_{i}=\frac{1}{n-1}\left[(n-2)\frac{{\overset{˙}{A}}_{1}}{A_{1}}+\frac{{\overset{˙}{A}}_{2}}{A_{2}}\right],\overset{¯}{A}=\frac{1}{n-1}\sum_{i=1}^{n-1}{\left(\frac{\Delta H_{i}}{H}\right)}^{2}=\frac{1}{n-1},$$

where a semicolon (;) followed by an index denotes partial differential. The deceleration parameter *q* which show that the model is decelerating.

Directional Hubble parameters (DPs) in the direction of $x_{n-2}$ and $x_{n-1}$ are obtained as

(2) $$H_{n-2}=(n-2)\frac{{\overset{˙}{A}}_{1}}{A_{1}},\;\;\;\;\;H_{n-1}=\frac{{\overset{˙}{A}}_{2}}{A_{2}}.$$

The shear scalar *σ* is defined as

$$\sigma^{2}=\frac{2}{3}\theta^{2},$$

where *θ* is the scalar expansion obtained as

$$\theta =u_{;i}^{i}=(n-1)H=(n-2)\left(\frac{{\overset{˙}{A}}_{1}}{A_{1}}\right)+\left(\frac{{\overset{˙}{A}}_{2}}{A_{2}}\right),$$

it's clear that the $\frac{\sigma }{\theta }$ is constant and hence it leads to an anisotropic model.

The relativistic field equations in f(R) theory of gravity are given by Hasmani et al. (2019) [19]

(3) $$G_{ij}=\frac{1}{f_{R}}\left[\frac{f(R)-Rf_{R}}{2}g_{ij}+G_{ij}+8\pi T_{ij}\right],\;\;i,j=1,2,3,4,$$

where

(4) $$G_{ij}=\nabla_{i}\nabla_{j}f_{R}-g_{ij}\nabla^{k}\nabla_{k}f_{R},=f_{RR}\left[R_{ij}-\Gamma_{ij}^{t}\overset{˙}{R}-g_{ij}\left(\overset{˙}{R}{\left(ln(\sqrt{-g})\right)}_{;t}\right)+\overset{¨}{R}\right]+f_{RRR}\left[R_{;i}R_{;j}-g_{ij}{\overset{˙}{R}}^{2}\right],$$

and

$$f_{R}\equiv \frac{df(R)}{dR},$$

*R* is the Ricci scalar, $R_{ij}$ is the Ricci tensor and $\nabla_{i}$ is the covariant derivative.

The energy momentum tensor (EMT) for cosmic string is given by

(5) $$T_{ij}=\rho u_{i}u_{j}-\lambda x_{i}x_{j},$$

where *ρ* is the rest density of energy cloud of strings, $u_{i}$ is 4-velocity of the fluid particles, *λ* is the tension density of the system of strings and $x_{i}$ is the unit space-like vector in the direction of anisotropy. The direction of string in the co-moving co-ordinate system will satisfy

(6) $$u_{n}u_{n}=-1=-x_{n-1}x_{n-1},\;\text{otherwise zero}.$$

The non-vanishing components $T_{ij}$ might be obtained as

(7) $$T_{nn}=-\rho ,\;T_{11}=T_{22}=...=T_{\left(n-2)(n-2\right)}=0,\;T_{\left(n-1)(n-1\right)}=-\lambda .$$

The field Equations (3) for the metric (1) with the assistance of (4), (5), (6) and (7) lead to the following system of equations

(8) $$(n-2)H_{1}H_{2}+\frac{\left(n-2)(n-3\right)}{2}H_{1}^{2}=\frac{1}{f_{R}}\left[\frac{f(R)-Rf_{R}}{2}-f_{RR}\left((n-2)H_{1}+H_{2}\right)-8\pi \rho \right],$$

(9) $$(n-2)\left({\overset{˙}{H}}_{1}+H_{1}^{2}\right)+\frac{\left(n-2)(n-3\right)}{2}H_{1}^{2}\;\;=\frac{1}{f_{R}}\left[\frac{f(R)-Rf_{R}}{2}-f_{RRR}-f_{RR}\left((n-2)H_{1}+H_{2}-8\pi \lambda \right)\right],$$

(10) $$(n-3)\left({\overset{˙}{H}}_{1}+H_{1}^{2}\right)+\left({\overset{˙}{H}}_{2}+H_{2}^{2}\right)+\frac{\left(n-3)(n-4\right)}{2}H_{1}^{2}+(n-3)H_{1}H_{2}\;\;=\frac{1}{f_{R}}\left[\frac{f(R)-Rf_{R}}{2}-f_{RRR}-f_{RR}\left((n-2)H_{1}+H_{2}\right)\right],$$

where $H_{1}=\frac{{\overset{˙}{A}}_{1}}{A_{1}}$ and $H_{2}=\frac{{\overset{˙}{A}}_{2}}{A_{2}}$.

## 3 Exact solutions of string cosmological model in f(R) theory of gravity

In this section, we find the string cosmological solutions of three independent field Equations (8) to (10) which connect five unknown quantities $A_{1},A_{2},\lambda ,\rho$ and f(R). Hence to get a determinate solution one has to assume physical or mathematical conditions. In the literature (Letelier (1983) [14], Takabayasi (1976) [24], Reddy (2003) [25]), the equations of state for string models are,

$$\rho =\left\{ \begin{array}{c} \lambda \;\;\text{(Geometry or Nambu string)},\\ (1+\omega )\lambda \;\;\text{(p-string)},\\ -\lambda \;\;\text{(Reddy string)}. \end{array}\right.$$

Since the field equations are highly non-linear, we also assume that the relation between metric coefficients $A_{2}=A_{1}^{m},\;m\neq 1$ where *m* is a an arbitrary constant. Let us consider the solution for f(R) gravity in the possible form of, i.e.,

$$f(R)=R+\alpha R^{2},$$

where *α* is real number. We shall now discuss the solution of above string models as *p* or Takabayasi string. Here the equation of state is

(11) ρ=(1+ω)λ

Now eliminating *ρ* and *λ* from (8) and (9) using (11) we obtain

(12) $$(n-1)[(m-\frac{\omega (n-3)}{2}){\left(\frac{\overset{˙}{A_{1}}}{A_{1}}\right)}^{2}-(\omega +1)\frac{\overset{¨}{A_{1}}}{A_{1}}]=\frac{\omega \alpha }{1+2\alpha R}\left(\frac{R^{2}}{2}+2(m+n-2)\frac{\overset{˙}{A_{1}}}{A_{1}}\right).$$

So for simplicity we take ω=0. Then from Equation (12) we get

(13) $$\frac{\overset{¨}{A_{1}}}{A_{1}}-m{\left(\frac{\overset{˙}{A_{1}}}{A_{1}}\right)}^{2}=0.$$

Integrating Equation (13), we obtain the following solutions for the directional scale factors $A_{1}$ and $A_{2}$

(14) $$A_{1}=c_{2}\left(-c_{1}-mt+t\right){}^{\frac{1}{1-m}},$$

(15) $$A_{2}=c_{2}^{m}\left(-c_{1}-mt+t\right){}^{\frac{m}{1-m}},$$

where $c_{1}$ and $c_{2}$ are integrating constants. The metric (1) in consideration with Equations (14) and (15) can be written as

(16) $$ds^{2}=dt^{2}-{\left(c_{2}\left(-c_{1}-mt+t\right){}^{\frac{1}{1-m}}\right)}^{2}\;\;\times \sum_{i=1}^{n-2}dx_{i}^{2}-{\left(c_{2}\left(-c_{1}-mt+t\right){}^{\frac{1}{1-m}}\right)}^{2m}dx_{n-1}^{2}.$$

The directional Hubble parameters in the direction of $x_{n-2}$ and $x_{n-1}$ as defined in (2) are found as

$$H_{n-2}=\frac{2-n}{(m-1)t+c_{1}},\;\;\;\;\;H_{n-1}=-\frac{m}{(m-1)t+c_{1}}.$$

The volume scale factor become

$$V=\left(c_{2}\left(-c_{1}-mt+t\right){}^{\frac{1}{1-m}}\right){}^{m+n-1}.$$

**Figure 1:**
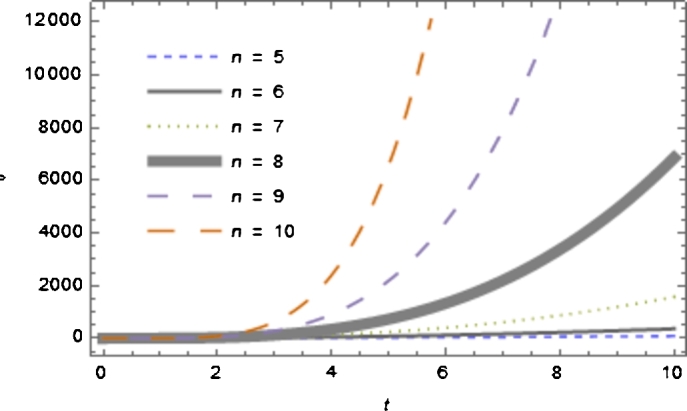
The plot of volume *V* versus cosmic time *t* with *m* = −1, *c*_1_ = *c*_2_ = 1 and different value of *n* dimensional.

The volume *V* is an increasing to infinite for large value of time *t* and is finite for *t* is finite. The model is a high dimensional string cosmological model where the volume increases whenever there is an increase of time representing the model is expanding. It means that the spatial volume *V* is constant at the beginning time of the universe t=0, it reaches in a finite time $t=\frac{-c_{1}}{m-1}$ to zero. (See Fig. 1.)

The solutions of *ρ* using Equation (8) simplifies to

(17) $$8\pi \rho =\left(1+2\alpha R\right)\left[H_{1}^{2}\left(m+\frac{\left(n-3\right)}{2}\right)\right]+\frac{\alpha R^{2}}{2}+2\alpha H_{1}R\left(n+m-2\right),$$

where

$$H_{1}=\frac{1}{-c_{1}-mt+t},$$

and the corresponding Ricci scalar is

$$R=\frac{\left(2m+n-3)(2m+n-2\right)}{{\left(c_{1}+(m-1)t\right)}^{2}}$$

For equation (17) we observed that ρ→0 as t→∞ (i.e., the rest energy *ρ* lends to zero as time increases indefinitely).

**Figure 2:**
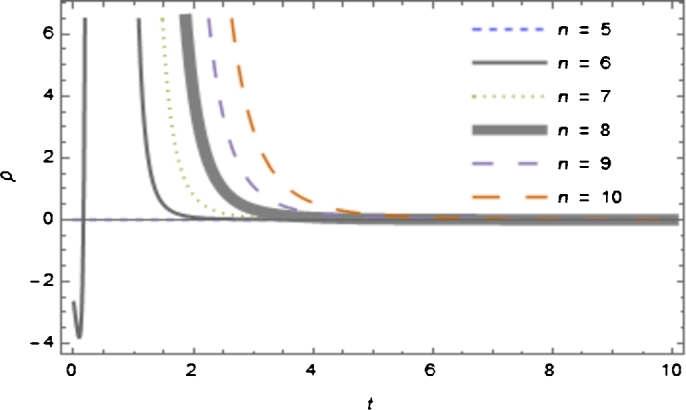
The plot of energy density *ρ* versus cosmic time *t* with *m* = −1, *α* = 2, *c*_1_ = *c*_2_ = 1 and different value of *n* dimensional.

From Fig. 2, it is observed that for n=5 and m=−1, the density of energy *ρ* vanishes, resulting in geometric string solution, i.e., ρ=0.

The kinematical quantities and physical solutions are obtained as

$$\theta =\frac{m+n-2}{c_{1}+(m-1)t},\sigma^{2}=\frac{2{\left(m+n-2\right)}^{2}}{3{\left(c_{1}+(m-1)t\right)}^{2}},q=\frac{1-mn}{m+n-2}.$$

From the above result, we observe that the physical parameters *θ* and $\sigma^{2}$ for the large *t* approached towards zero. It has singularity at $t=\frac{-c_{1}}{m-1}$. Hence, the physical parameters *θ* and $\sigma^{2}$ diverges at $t=\frac{-c_{1}}{m-1}$.

The physical parameters *θ* and $\sigma^{2}$ evolve with time in between the Big Bang and $t=\frac{-c_{1}}{m-1}$. The model of the universe starts with constant and ends with $t=\frac{-c_{1}}{m-1}$. It is decrease as time *t* gradually increases, and finally they vanish when t→∞. Since,

$$\lim_{t\to \infty }\frac{\sigma^{2}}{\theta }=0,$$

so the model approaches highly isotropy for large value of cosmic time *t*.

Also, m≠1 is an arbitrary constant, the deceleration parameter is negative for m>1 which implies that the model (16) accelerating expansion if −1<q<0 (also known as power-law expansion), de Sitter expansion for q=−1 (also known as exponential expansion) and super-exponential for q<−1, the deceleration parameter is positive for m<1, so the universe exhibit decelerating expansion. These DP, correspond to Berman's law of constant deceleration parameter, see Berman (1983) [26], Sahoo et al. (2017) [27] for more details.

## 4 Conclusion

Some exact solutions of the field equations are obtained for the higher dimensional Kaluza-Klein universe with string cosmological models in the frame work of f(R) theory of gravity. The solution obtained using the EoS for Nambu string by (ρ=λ), (i.e., geometry string or Nambu string). For the models (16), the directional Hubble parameters are finite at t=0 and approach zero monotonically at t→∞. Also the scale of expansion *θ* is finite at t=0 and θ→0 when t→∞ whereas ρ→0 as t→∞, thus the models tends empty universe when t→∞ and the model approaches isotropy, which gives better clarity on the accelerated expansion of the universe.

## Declarations

### Author contribution statement

Ahmed M. Alhaysah: Conceived and designed the analysis; Analyzed and interpreted the data; Wrote the paper.

A.H. Hasmani: Analyzed and interpreted the data; Wrote the paper.

### Declaration of interests statement

This research did not receive any specific grant from funding agencies in the public, commercial, or not-for-profit sectors.

### Data availability statement

Data included in article/supplementary material/referenced in article.

### Funding statement

The authors declare no conflict of interest.

### Additional information

No additional information is available for this paper.
